# Adaptation to a Commercial Quaternary Ammonium Compound Sanitizer Leads to Cross-Resistance to Select Antibiotics in *Listeria monocytogenes* Isolated From Fresh Produce Environments

**DOI:** 10.3389/fmicb.2021.782920

**Published:** 2022-01-10

**Authors:** Rebecca Bland, Joy Waite-Cusic, Alexandra J. Weisberg, Elizabeth R. Riutta, Jeff H. Chang, Jovana Kovacevic

**Affiliations:** ^1^Food Innovation Center, Oregon State University, Portland, OR, United States; ^2^Department of Food Science and Technology, Oregon State University, Corvallis, OR, United States; ^3^Department of Botany and Plant Pathology, Oregon State University, Corvallis, OR, United States

**Keywords:** antibiotic resistance, cross-resistance, quaternary ammonium compound, sanitizers, whole genome sequencing

## Abstract

The effective elimination of *Listeria monocytogenes* through cleaning and sanitation is of great importance to the food processing industry. Specifically in fresh produce operations, the lack of a kill step requires effective cleaning and sanitation to mitigate the risk of cross-contamination from the environment. As facilities rely on sanitizers to control *L. monocytogenes*, reports of the development of tolerance to sanitizers and other antimicrobials through cross-resistance is of particular concern. We investigated the potential for six *L. monocytogenes* isolates from fresh produce handling and processing facilities and packinghouses to develop cross-resistance between a commercial sanitizer and antibiotics. Experimental adaptation of isolates belonging to hypervirulent clonal complexes (CC2, CC4, and CC6) to a commercial quaternary ammonium compound sanitizer (cQAC) resulted in elevated minimum inhibitory concentrations (2–3 ppm) and minimum bactericidal concentrations (3–4 ppm). Susceptibility to cQAC was restored for all adapted (qAD) isolates in the presence of reserpine, a known efflux pump inhibitor. Reduced sensitivity to 7/17 tested antibiotics (chloramphenicol, ciprofloxacin, clindamycin, kanamycin, novobiocin, penicillin, and streptomycin) was observed in all tested isolates. qAD isolates remained susceptible to antibiotics commonly used in the treatment of listeriosis (i.e., ampicillin and gentamicin). The whole genome sequencing of qAD strains, followed by comparative genomic analysis, revealed several mutations in *fepR*, the regulator for FepA fluoroquinolone efflux pump. The results suggest that mutations in *fepR* play a role in the reduction in antibiotic susceptibility following low level adaptation to cQAC. Further investigation into the cross-resistance mechanisms and pressures leading to the development of this phenomenon among *L. monocytogenes* isolates recovered from different sources is needed to better understand the likelihood of cross-resistance development in food chain isolates and the implications for the food industry.

## Introduction

*Listeria monocytogenes* is a Gram-positive bacterium that remains one of the leading causes of mortality among foodborne pathogens in the United States (Scallan et al., 2011; de Noordhout et al., 2014). Listeriosis, the disease caused by *L. monocytogenes*, largely effects vulnerable populations, including pregnant women, children, elderly, and people who are immunocompromised (Hamon et al., 2006; Buchanan et al., 2017). Due to the immune status of this vulnerable population and the invasive nature of the disease, antibiotics are critical to the successful treatment of listeriosis. The first choice antibiotic for the treatment is typically ampicillin (ß-lactam), alone or in combination with gentamicin (aminoglycoside) (Hof, 2004). While *L. monocytogenes* remains largely susceptible to a wide range of antibiotics, there have been reports of multidrug resistance in isolates recovered from food production environments (Prazak et al., 2002; Jorgensen et al., 2021).

*Listeria monocytogenes* is well adapted to agricultural environments, where it proliferates in decaying plant matter in the soil (Linke et al., 2014; Liao et al., 2021). It is also frequently recovered from diverse food processing environments (Gray et al., 2006; Freitag et al., 2009; Cherifi et al., 2018; Kim et al., 2018; Hurley et al., 2019; Sullivan and Wiedmann, 2020). *Listeria monocytogenes* prevalence in these environments presents a particular concern to processors of minimally processed ready-to-eat (RTE) products, such as fresh produce, that are prone to contamination during handling and processing (Gallagher et al., 2003; Farber et al., 2020). Often, control of *L. monocytogenes* in these environments relies on cleaning and sanitation programs. In the United States, sanitizers are regulated by the Environmental Protection Agency (EPA) and are required to achieve a 5-log reduction of a test organism on a food contact surface and a 3-log reduction on a non-food contact surface (Office of Chemical Safety and Pollution Prevention [OCSPP], 2012). Sanitizers are formulated at concentrations that will deliver a bactericidal effect, and in general many-fold higher than the minimum bactericidal concentration (MBC) for foodborne pathogens of concern (Cruz and Fletcher, 2012).

Quaternary ammonium compounds (QACs) are one group of sanitizers commonly used in the food industry. QACs are cationic antimicrobials with medium-to-long alkyl side chains (Gilbert and Moore, 2005; Wessels and Ingmer, 2013). The mechanism of bacterial cell inhibition is generally thought to be by the hydrophobic chain interpolating into the lipid bilayer of the cellular membrane leading to issues in osmoregularity and leaking of cell contents. The manufacturer recommended concentrations (MRCs) for commercial QAC (cQAC) application range from 200 to 800 ppm (Cruz and Fletcher, 2012; Boucher et al., 2021). The reported minimum inhibitory concentrations (MICs) for *L. monocytogenes* to QACs range from ≤2 ppm (Romanova et al., 2006; Martínez-Suárez et al., 2016; Møretrø et al., 2017; Yu et al., 2018; Roedel et al., 2019) to 40 ppm (Elhanafi et al., 2010; Dutta et al., 2013). Ideally, appropriate cleaning and sanitation procedures are paired with optimum hygienic design of the facility and equipment; however, this ideal combination is rare, particularly in fresh produce facilities. These less-than-ideal environments can create scenarios where *L. monocytogenes* could be exposed to sublethal sanitizer concentrations. Links between reduced susceptibility to QACs and antibiotic resistance have been reported for various foodborne bacteria, including *L. monocytogenes* (Heir et al., 1999; Braoudaki and Hilton, 2004; Langsrud et al., 2004; Rakic-Martinez et al., 2011; Gnanadhas et al., 2013; Kovacevic et al., 2013; Bansal et al., 2018; Kode et al., 2021). This phenomenon of cross-resistance can occur when microorganisms develop survival methods that are effective against different antimicrobial agents with similar mechanisms of action (SCENIHR, 2009). For example, a mutation or upregulation of a gene initiated by adaptation or exposure to one of the antimicrobials may subsequently affect the efficacy of another antimicrobial agent (Jiang et al., 2018; Amsalu et al., 2020).

We previously evaluated the prevalence and distribution of antimicrobial resistance (AMR) in *L. monocytogenes* from produce handling and processing facilities in the Pacific Northwest (Jorgensen et al., 2021). These isolates have diverse antibiogram profiles and represent clonal complexes associated with hypervirulent phenotypes. The present study investigated the potential for antibiotic cross-resistance to develop in these strains following adaptation to a commercial sanitizer (cQAC). Culture-based assays and whole genome sequence (WGS) comparisons were used to evaluate differences between the wild-type (WT) and cQAC-adapted (qAD) strains.

## Materials and Methods

### Bacterial Strains and Sanitizer

A total of six *L. monocytogenes* isolates previously recovered from produce packing, processing, and handling environments in the Pacific Northwest (Jorgensen et al., 2020, 2021) were selected for adaptation to cQAC (Table 1). All isolates were previously serogrouped, multi-locus sequence typed and assessed for AMR using a standard disk diffusion assay (CLSI, 2015; Jorgensen et al., 2021). Isolates used in this study were selected based on their AMR profiles and multi-locus sequence types (MLST). Specifically, up to two isolates belonging to each available hypervirulent clonal complex (CC) 2, CC4, and CC6 (Maury et al., 2016), and with unique AMR profiles (when available) were included in this study. Isolates were stored at –80°C in trypticase soy broth (TSB; Acumedia, Neogen, Lansing, MI, United States) with 25% (v/v) glycerol. Prior to use, isolates were resuscitated on trypticase soy agar (TSA; Acumedia) with incubation at 35°C for 24 h and used for a maximum of 2 weeks.

**TABLE 1:** Genetic profiles and characterization of *Listeria monocytogenes* isolates (*n* = 6) selected for evaluation of cross-resistance.

Isolate no.	Sequence type	Clonal complex	LIPI-3	LIPI-4	*inlA* *^a^*
WRLP354	2	2	−	−	+
WRLP380	2	2	−	−	+
WRLP394	219	4	+	+	+
WRLP483	219	4	+	+	+
WRLP530	6	6	+	−	3-codΔ
WRLP533	6	6	+	−	3-codΔ

*All strains were isolated from produce operations in the Pacific Northwest during 2018–2019 by Jorgensen et al. (2020).*

*^a^inlA 3-codΔ indicates a 3-codon deletion in amino acid positions 738–740 (aspartic acid, threonine, and serine).*

The sanitizer used in this study was a commercial quaternary ammonium compound, cQAC (1–6 ppm; Professional Lysol No Rinse Sanitizer; EPA registration 675-30; Reckitt Benckiser, Parsippany, NJ, United States). Stock solution of the cQAC sanitizer was prepared in accordance with the manufacturer recommended concentration (MRC; 200 ppm), filter sterilized, and stored for up to 1 week at 4°C.

### Minimum Inhibitory and Minimum Bactericidal Concentration

Microbroth dilution assay described by Boucher et al. (2021) was used to assess minimum inhibitory (MICs) and minimum bactericidal concentration (MBCs) of cQAC, with minor modifications. Briefly, a single colony was transferred to TSB (5 ml) and incubated at 30°C for 16 h, with shaking (150 rpm; Thermo Scientific, MaxQ4000, Waltham, MA, United States). Following incubation, each culture was diluted to approximately 7 log CFU/ml in 0.1% peptone water (Fisher; Hampton, NH, United States). Inoculum was confirmed on TSA incubated at 35°C for 24 h using the track dilution method by Jett et al. (1997). Diluted cultures were added to TSB with 0.6% yeast extract (TSB-YE; Acumedia) containing 1, 2, 3, 4, 5, or 6 ppm cQAC (i.e., diluted from MRC stock solution) at approximately 5 log CFU/ml in a final volume of 10 ml. An aliquot (200 μl) of each culture/sanitizer mixture was transferred to a sterile 96-well plate (VWR; Radnor, PA, United States), in duplicate. Plates were incubated at 30°C in a SpectraMax plate reader (Molecular Devices). OD_600_ was measured at 30 min interval for 24 h with 5 s of shaking prior to measurement. The OD_600_ data were fitted to growth curves to obtain the lag-phase duration (LPD), maximum growth rate (MGR), and maximum density, using the DMFit 3.0 Excel add-in program (ComBase; Computational Microbiology Research Group, Institute of Food Research, Colney, Norwich, United Kingdom), based on the models of Baranyi and Roberts (1994). Growth curve experiments were performed at least three times. For each isolate a cut off value of 0.1 maximum OD_600_ was used to define inhibition. The lowest concentration with a max OD_600_ of <0.1 was interpreted as the MIC. In addition, MICs of WT and qAD strains were tested in the presence of reserpine (Alfa Aesar, Tewksbury, MA, United States), a known efflux inhibitor (Godreuil et al., 2003; Kovacevic et al., 2016). A working solution of reserpine (1,000 μg/ml) was prepared in dimethyl sulfoxide (DMSO; VWR) prior to MIC assay and stored at 4°C for 24 h. Uninoculated sanitizer and sterile media controls were included in each replicate and their OD_600_ values served as the baseline for the sanitizer treatment. Following 24 h incubation in the plate reader, each well was streaked onto TSA with 0.6% yeast extract (TSA-YE; Acumedia) and incubated at 35°C for 24 h. The lowest cQAC concentration where no growth was observed was considered the MBC.

Minimum inhibitory concentration of all WT and qAD isolates for ciprofloxacin was also measured using the VITEK system (Biomerieux, France; card: AST-GP75) according to manufacturer instructions.

### Experimental Adaptations to Sublethal Concentrations of Sanitizers

**FIGURE 1:**
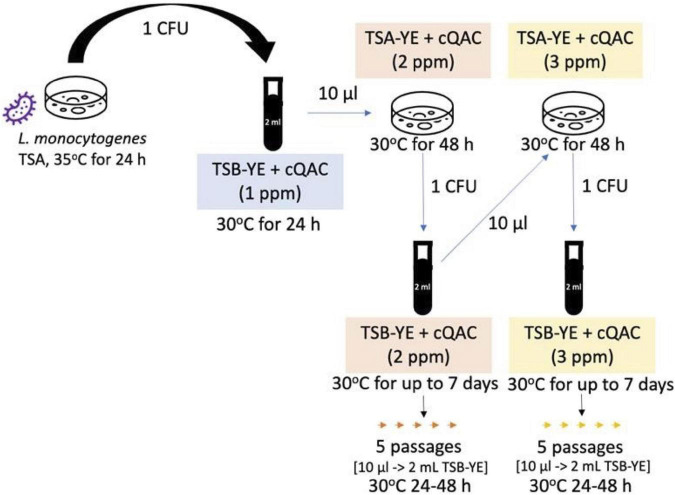
Experimental scheme used to adapt *L. monocytogenes* to cQAC.

*Listeria monocytogenes* strains were experimentally adapted to increasing concentrations of cQAC sanitizer. Strains were sub-cultured at 30°C with progressively higher concentrations of cQAC, alternating between TSB-YE (2 ml, 150 rpm shaking), and TSA-YE plates (Figure 1). Adaptations started in TSB-YE at a concentration of 1 ppm. Once the culture was visibly turbid, 10 μl was transferred into fresh TSB-YE and TSA-YE media with cQAC (2 ml total volume for TSB-YE and 15 ml for TSA-YE plates). Concentration of cQAC present in the media was increased by 1 ppm following stabilization of each incremental adaptation in TSB-YE. Once growth was observed on TSA-YE, a single colony was transferred to TSB-YE at the same concentration to stabilize the adaptation at each increment. Adaptations were stopped when no growth was visually observed after 5 days of incubation at 30°C in TSB-YE. The adaptations scheme used in the isolates reported here is depicted in Figure 1. For each cQAC concentration adaptation (1, 2, and 3 ppm), strains were stabilized by five passages in TSB-YE with appropriate cQAC concentration. Adapted strains were frozen in TSB supplemented with cQAC at half the adapted concentration with 25% (v/v) glycerol. TSA-YE supplemented with 3 ppm cQAC was used to revive cQAC-adapted cultures from frozen stock for use in the following assays.

### Whole Genome Sequencing of Commercial Quaternary Ammonium Compound Adapted Isolates

Commercial quaternary ammonium compound adapted *L. monocytogenes* isolates (qAD) were revived from frozen stock on TSA-YE supplemented with 3 ppm cQAC and incubated at 35°C for 24 h. A single colony was transferred to 3 ml TSB and incubated at 35°C for 18–20 h with shaking (150 rpm). DNA was extracted using Qiagen Blood and Tissue kit (Qiagen, Germantown, MD, United States) according to the manufacturer recommendations for Gram-positive bacteria. Whole genome sequencing (WGS) libraries were prepared with the Nextera XT DNA sample preparation kit (Illumina, San Diego, CA, United States), according to the manufacturer’s protocol. Paired-end sequencing (2 × 150 bp) was performed on the Illumina MiSeq by the Center for Quantitative Life Sciences (CQLS, Oregon State University, Corvallis, OR, United States). Raw sequence reads were quality checked with FastQC, followed by quality trimming with Trimmomatic (v 0.39). Reads were *de novo* assembled using SPAdes optimized with unicycler (v 0.4.8) and assemblies were annotated with Prokka (v 1.14.6). Mutations were identified by mapping the reads of the adapted isolates to draft assemblies of the respective wild types. Briefly, raw reads were mapped to the respective reference sequence using BWA (v 0.7.17). Alignments were annotated, sorted and duplicate reads identified with Picard tools (v 2.0.1). Graphtyper (v 2.6.2) was run on each dataset with the default parameters (Eggertsson et al., 2017). Single nucleotide polymorphisms (SNP) were filtered using vcffilter in vcflib (v 1.0.0). SNP calls annotated as “FAIL” or “heterozygous” were filtered to “no-call”. SnpEff (v 4.3t) with the parameters “-no-downstream -no-upstream -no-intron” was used to predict functional effects of each SNP (Cingolani et al., 2012). Geneious (v 2020.1.2) was used to further investigate mutations and genes with identified mutations were aligned using MUSCLE (Edgar, 2004). Sequences for adapted and WT isolates are available under SRA BioProject PRJNA771688.

### Antibiotic Susceptibility Disk Diffusion Assay

Following sanitizer adaptations, disk diffusion assays were used to determine if sanitizer adaptations affected sensitivity to a panel of 17 antibiotics (BBL Sensi-Disc, BD Diagnostics, Sparks, MD, United States). The tested antibiotic disks included: amikacin (AMK; 30 μg), ampicillin (AMP; 10 μg), cefoxitin (FOX; 30 μg), chloramphenicol (CHL; 30 μg), ciprofloxacin (CIP; 5 μg), clindamycin (CLI; 2 μg), erythromycin (ERY; 15 μg), gentamicin (GEN; 10 μg), kanamycin (KAN; 30 μg), novobiocin (NOV; 30 μg), penicillin G (PEN; 10 μg) rifampicin (RIF; 5 μg), streptomycin (STR; 10 μg), cotrimoxazole (SXT; 1.25/23.75 μg), imipenem (IMP; 10 μg) tetracycline (TET; 30 μg), and vancomycin (VAN; 5 μg). Disk diffusion assays were carried out as described by Jorgensen et al. (2021). The diameter of each zone of inhibition was measured to the nearest mm. Interpretation of antibiotic susceptibility (sensitive, intermediate, and resistant) was determined in accordance with the Clinical Laboratory Standards Institute criteria (CLSI, 2015) and compared to the measurements and susceptibility classifications established by Jorgensen et al. (2021) for each tested isolate (Supplementary Table 1). *Listeria monocytogenes* isolates displaying resistance to specific antibiotics were confirmed with up to two disk diffusion assays. In all assays *Escherichia coli* ATCC 35218 and *Staphylococcus aureus* ATCC 25923 were used as control strains. All results were compared with previously determined zones of inhibition for the WT strains described by Jorgensen et al. (2021).

### Statistical Analysis

Comparisons between WT and qAD isolates within a treatment was performed using an unpaired, two-tail *t* test, while a comparison between a treatment (e.g., TSB-YE + R, or 2 ppm cQAC) and control (TSB-YE) for WT or qAD isolates was performed using a paired two-tailed *t* test in Excel. For all analyses, differences were considered significant if the *P* value was <0.05.

## Results

### Commercial Quaternary Ammonium Compound Adaptations for *Listeria monocytogenes*

All six *L. monocytogenes* isolates were successfully adapted to tolerate an additional 1 ppm of cQAC as determined by MIC (increased from 2 to 3 ppm) and MBC (increased from 3 to 4 ppm) assays (Table 2). Further attempts to adapt to higher concentrations of cQAC were not successful. In the presence of reserpine, a known efflux pump inhibitor, the MIC and MBC of all qAD strains were identical to the non-adapted WT strains. Reserpine had no effect on the MBC of WT strains; however, the WT strains were no longer able to grow in the presence of 1 ppm of cQAC (lowest concentration tested).

**TABLE 2:** Minimum inhibitory concentrations (MIC) and minimum bactericidal concentrations (MBC) of a commercial quaternary ammonium compound sanitizer (cQAC) for wild-type (WT) and cQAC-adapted (qAD; 3 ppm) *L. monocytogenes* strains in the absence or presence of reserpine (+R).

Isolate no.	Concentration (ppm)^a^			
	WT^b^	WT + R^c^	qAD	qAD + R
WRLP354				
MIC^b^	2	<1	3	2
MBC^b^	3	3	4	3
WRLP380				
MIC	2	<1	3	2
MBC	3	3	4	3
WRLP394				
MIC	2	<1	3	2
MBC	3	3	4	3
WRLP483				
MIC	2	<1	3	2
MBC	3	3	4	3
WRLP530				
MIC	2	<1	3	2
MBC	3	3	4	3
WRLP533				
MIC	2	<1	3	2
MBC	3	3	4	3

*^a^Manufacturer recommended concentration (MRC) for the cQAC is 200 ppm.*

*^b^Reported MIC and MBC tested in stepwise increments over three biological replicates.*

*^c^In the presence of reserpine, WT isolates did not grow at the lowest dose of 1 ppm cQAC.*

Growth properties of WT and qAD *L. monocytogenes* strains in TSB-YE with or without cQAC (2 ppm) and/or reserpine (20 μg/ml) are described in Table 3. All WT and qAD strains were comparable in lag phase duration (LPD; h) and maximum cell density (OD_600_) when grown in standard TSB-YE (*P* > 0.05). One qAD strain (WRLP354) had a significantly slower (*P* < 0.05) maximum growth rate in TSB-YE (0.13 ± 0.01 OD_600_/h) when compared to its respective WT strain (0.17 OD_600_/h) (Table 3, indicated by *). The addition of reserpine in TSB-YE led to a slight increase in the LPD for both WT (1.62–2.32 h) and qAD (1.17–2.45 h) strains, though this was not statistically significant. Reserpine did not seem to impact the growth rate of the majority of WT and qAD isolates. The only exception were WT WRLP354 and WRLP394 strains, which grew slower in the presence of reserpine compared to their growth in TSB-YE (*P* < 0.05; Table 3, indicated by #). There was no statistical difference between maximum OD_600 *nm*_ of WT and qAD strains (Table 3).

**TABLE 3:** Average lag phase duration, maximum growth rate, and maximum optical density of wild type (WT) and cQAC-adapted (qAD) *L. monocytogenes* strains exposed to sublethal concentration of cQAC (2 ppm) in tryptic soy broth with yeast extract (TSB-YE), with and without reserpine (R; 20 μg/ml), at 30°C for 24 h.

Isolate and treatment	Lag-phase duration (h)*^a,b^*		Maximum growth rate (increase in OD_600_/h)*^a,c^*		Maximum OD_600_*^a^*	
	WT*^d^*	qAD*^e^*	WT	qAD	WT	qAD
WRLP354						
TSB-YE	8.43 ± 0.36	8.95 ± 0.19	0.17 ± 0.00	0.13 ± 0.01^*^^a^	0.59 ± 0.02	0.56 ± 0.03
TSB-YE + R*^f^*	10.49 ± 0.06	10.53 ± 0.05	**0.12 ± 0.01^#*c*^**	0.12 ± 0.01	0.49 ± 0.07	0.49 ± 0.06
2 ppm cQAC	–*^g^*	10.50 ± 1.30	–	0.14 ± 0.01	–	0.50 ± 0.01
2 ppm cQAC + R	–	–	–	–	–	–
WRLP380						
TSB-YE	8.34 ± 0.38	8.37 ± 0.43	0.16 ± 0.01	0.12 ± 0.02	0.60 ± 0.03	0.60 ± 0.03
TSB-YE + R	9.96 ± 0.46	10.32 ± 0.38	0.13 ± 0.01	0.13 ± 0.01	0.52 ± 0.03	0.52 ± 0.03
2 ppm cQAC	–	10.48 ± 1.86	–	0.13 ± 0.02	–	0.55 ± 0.0
2 ppm cQAC + R	–	–	–	–	–	–
WRLP394						
TSB-YE	8.08 ± 0.40	8.01 ± 1.09	0.17 ± 0.01	0.11 ± 0.03	0.65 ± 0.02	0.67 ± 0.02
TSB-YE + R	10.40 ± 0.19	10.46 ± 0.16	**0.14 ± 0.01^#^**	0.14 ± 0.01	0.54 ± 0.04	0.54 ± 0.03
2 ppm cQAC	–	9.95 ± 2.48	–	0.13 ± 0.04	–	0.59 ± 0.03
2 ppm cQAC + R	–	–	–	–	–	–
WRLP483						
TSB-YE	8.24 ± 0.40	8.41 ± 0.34	0.16 ± 0.01	0.15 ± 0.02	0.59 ± 0.00	0.56 ± 0.03
TSB-YE + R	9.85 ± 0.23	9.58 ± 0.19	0.13 ± 0.02	0.13 ± 0.02	0.48 ± 0.02	0.48 ± 0.01
2 ppm cQAC	–	**10.41 ± 0.25^$b^**	–	0.15 ± 0.02	–	0.50 ± 0.02
2 ppm cQAC + R	–	–	–	–	–	–
WRLP530						
TSB-YE	8.58 ( 0.25	9.04 ( 0.16	0.16 ( 0.01	0.14 ( 0.01	0.64 ( 0.03	0.59 ( 0.04
TSB-YE + R	10.29 ± 0.37	10.07 ± 0.30	0.13 ± 0.02	0.13 ± 0.01	0.52 ( 0.01	0.52 ( 0.01
2 ppm cQAC	–	**11.66 ± 0.11^$^**	–	0.14 ± 0.02	–	0.53 ± 0.00
2 ppm cQAC + R	–	–	–	–	–	–
WRLP533						
TSB-YE	8.41 ( 0.41	8.99 ( 0.12	0.16 ( 0.01	0.14 ( 0.01	0.65 ( 0.02	0.61 ( 0.06
TSB-YE + R	10.62 ± 0.23	10.73 ± 0.19	0.13 ± 0.02	0.13 ± 0.01	0.51 ± 0.02	0.51 ± 0.01
2 ppm cQAC	–	**10.98 ± 0.32^$^**	–	0.12 ± 0.05	–	0.54 ± 0.03
2 ppm cQAC + R	–	–	–	–	–	–

^a^Values represent mean values ± standard deviation for three independent assays, with each sample and treatment measured in duplicate. Statistically significant values between WT and qAD strains within each treatment are indicated by asterisk (*P < 0.05, unpaired two-tailed t test).

*^b^Statistically significant values of lag phase duration of qAD strains grown in the presence of 2 ppm cQAC compared to TSB-YE are indicated by $ (P < 0.05, paired two-tailed t test).*

*^c^Statistically significant values of maximum growth rate of WT strains grown in TSB-YE compared to TSB-YE + R are indicated by # (P < 0.05, paired two-tailed t test).*

*^d^WT, wild type.*

*^e^qAD, strains adapted to 3 ppm cQAC, a commercial quaternary ammonium compound sanitizer.*

*^f^R, reserpine added at 20 μg/ml concentration.*

*^g^No growth is indicated by “–”.*

*The bold values represent statistically significant results.*

The addition of 2 ppm cQAC (equivalent to MIC) to TSB-YE inhibited the growth of all WT *L. monocytogenes* strains. All qAD strains grew under these conditions; however, the LPD was significantly longer (1.55–2.62 h; *P* < 0.05) in 3/6 isolates (WRLP483, WRLP530, and WRLP533; Table 3, indicated by $) compared to their growth in TSB-YE (i.e., without cQAC). However, there were no significant differences in maximum OD_600 *nm*_ for any of the tested isolates when grown in the presence of 2 ppm cQAC compared to TSB-YE. None of the qAD strains were able to grow in the presence of 2 ppm cQAC and reserpine. Two of the qAD strains, both representing CC2 (WRLP354 and WRLP380), were capable of growth in the presence of 3 ppm cQAC, with LPD > 19.5 h (data not shown). However, when both strains were exposed to 3 ppm cQAC with reserpine, their growth was inhibited.

### Antibiotic Susceptibility of Commercial Quaternary Ammonium Compound-Adapted Strains

**FIGURE 2:**
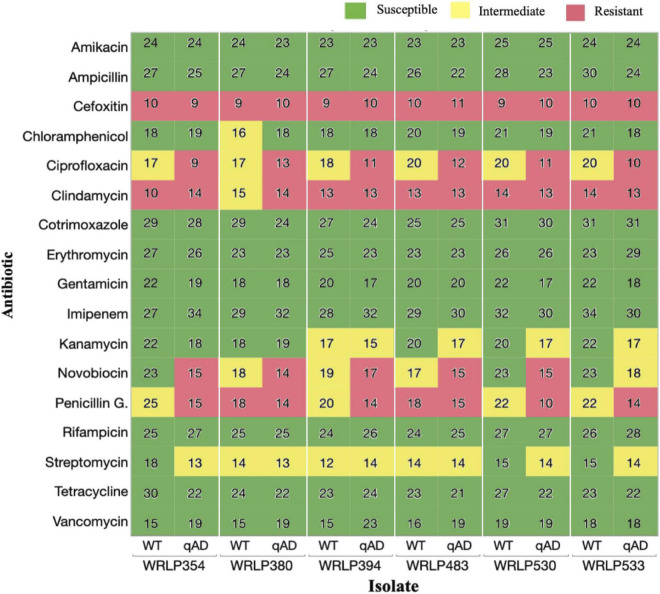
Antibiotic susceptibility of wild-type (WT) and cQAC adapted (qAD) *Listeria monocytogenes* strains (*n* = 6) to 17 antibiotics. Values reported represent zone diameters measured in mm. For adapted isolates, the median of 2–3 independent replicates is reported. Susceptibility (green), intermediate resistance (yellow), and resistance (red) classifications, as determined by the CLSI standards for *L. monocytogenes* and previously reported literature, are illustrated by different colors.

Isolates adapted to cQAC (qAD) over five serial passages resulted in changes in antibiotic susceptibility to 7/17 antibiotics tested, including CHL, CIP, CLI, KAN, NOV, PEN, and STR (Figure 2). All six qAD isolates resulted in varying degrees of antibiogram profile changes, as seen in the color changes between WT and qAD isolates in Figure 2. However, no obvious difference in cross-resistance patterns was seen among the three CCs evaluated.

Following adaptation to cQAC, no isolate appeared to have more advantage in the development of cross-resistance toward tested antibiotics. Isolates WRLP530 and WRLP533 exhibited changes in susceptibility to 5/17 antibiotics; followed by WRLP354, which exhibited changes in susceptibility to 4/17 antibiotics; while shifts in AMR profiles for 3/17 antibiotics were seen in isolates WRLP380, WRLP394, and WRLP483 (Figure 2).

All WT isolates possessing intermediate resistance to PEN (4/17) resulted in reduced susceptibility and shift in resistance classification, based on the zone of inhibition, following adaptation to cQAC (Figure 2). With the exception of one isolate (WRLP380), all WT strains possessed resistance to CLI. Following adaptation to cQAC, WRLP380 profile changed from intermediate to resistant to CLI. Similarly, all WT isolates (6/6) that initially had intermediate resistance to CIP, became resistant following cQAC adaptation (Figure 2). Due to the consistency in profile changes across the isolates for CIP, the MIC was measured to confirm the changes observed with the disk diffusion. Isolate pairs (WT and qAD) had MIC differences ranging from 0.5 to 2 μl/ml and 1 to >8 μl/ml (Table 4).

**TABLE 4:** Minimum inhibitory concentrations (MIC) of ciprofloxacin for wild-type (WT) and cQAC-adapted (qAD; 3 ppm cQAC) *L. monocytogenes* strains.

Isolate no.	Concentration CIP (μl/ml)	
	WT	qAD
WRLP354	1	>8
WRLP380	<0.5	2
WRLP394	1	>8
WRLP483	1	>8
WRLP530	1	4
WRLP533	1	4

In contrast, varying degrees of susceptibility shifts were seen amongst isolates for NOV before and after cQAC adaptations. Of the antibiotics where cross-resistance was observed, NOV was the only one to have WT classifications in the susceptible, intermediate, and resistant category. Following adaptation, isolates initially susceptible (WRLP354 and WRLP530) and intermediate (WRLP380 and WRLP394, WRLP483) to NOV became resistant. None of the isolates evaluated possessed resistance to STR or CHL prior to or following cQAC adaptation. While there was a reduction in susceptibility for STR in 3/6 isolates following adaptation, only one adapted isolate resulted in a susceptibility change in CHL (WRLP380; Figure 2). In contrast to other susceptibility changes, this shift resulted in increased susceptibility to CHL for WRLP380.

### Genomic Analysis to Identify Differences in Wild-Type and Commercial Quaternary Ammonium Compound-Adapted *Listeria monocytogenes*

Comparisons of whole genome sequences of each qAD and WT pair revealed between 0 and 4 confidently called deletions and SNPs predicted to reflect mutations. Some of these SNPs were found to be in non-coding regions within the genome (Table 5). Notably, SNPs in the *fepR* gene were seen in 5/6 isolates. This gene is predicted to encode a transcriptional regulator of FepA, a multidrug efflux pump. In three isolates with *fepR* mutations (WRLP380, WRLP483, and WRLP533), a SNP is predicted to cause a change in an amino acid. In WRLP394 and WRLP483, the SNP is predicted to result in a premature stop codon (PMSC) at position 141 and 175, respectively, truncating the protein by 55 and 21 amino acids, respectively. Deletions ranging from 1 to 33 bp were identified in the other three isolates (WRLP394, WRLP530, and WRLP533). The largest deletion (33 bp) is in WRLP530; the only genetic alteration observed in this isolate. Notably, no two isolates had a deletion at the exact same location within the *fepR* gene (Table 5).

**TABLE 5:** Mutations in *L. monocytogenes* isolates following the adaptation to commercial quaternary ammonium compound-based sanitizer (cQAC; 3 ppm).

Isolate no.	Total # high quality mutations	Transcriptional regulator *fepR*		
		Mutation	AA change	Nucleotide position
WRLP354	n/m*^a^*			
WRLP380	2	G > A*^b^*	Pro > Ser	322
WRLP394	4	1 nt deletion*^c^* A > T G > T	Leu > PMSC	288 291 292
WRLP483	2	C > A*^b^* G > T*^c^*	Pro > Gln Glu > PMSC	320 526
WRLP530	3	33 nt deletion		40
WRLP533	2	C > T*^b^* 6 nt deletion	His > Tyr	121 126–131

*^a^n/m, no mutation.*

*^b^Mutation resulted in a change in amino acid (AA) sequence.*

*^c^Mutation resulted in a premature stop codon (PMSC).*

## Discussion

Produce processing facilities, which have frequent turnover of raw agricultural products, present a unique risk for *L. monocytogenes* contamination. In these environments, *L. monocytogenes* is largely controlled through effective cleaning and sanitation practices. Previous reports of sanitizer and antibiotic cross-resistance have proposed broader consequences for lapses in sanitation efficacy in the food industry. In the present study, we demonstrated the ability of *L. monocytogenes* strains, including those representing hypervirulent clonal complexes, to adapt and tolerate slightly elevated concentrations of a commercial QAC (MIC 3 ppm; MBC 4 ppm) compared to the WT strains.

The six *L. monocytogenes* isolates evaluated were adaptable to a commercial QAC at 1 ppm above their WT MIC (2 ppm) and MBC (3 ppm). Previous studies using benzalkonium chloride (BC) compound noted a greater increase in MIC following adaptations. Similar to WT strains in the present study, Aase et al. (2000) reported five *L. monocytogenes* isolates with an initial MIC of 2 ppm; however, these isolates were then adapted to MICs of 6–7 ppm. It is of interest to note that other isolates tested within their study, with an initial MIC of 6 and 7 ppm, did not exceed 7 ppm MIC following the adaptation process (Aase et al., 2000). This suggests a potential plateau at which isolates are no longer readily adaptable, as we have observed in our study. Similar to Aase et al. (2000), a study by To et al. (2002) reported BC adaptation of *L. monocytogenes* isolates from 1 or 4 ppm MIC to 6 or 8 ppm MIC, respectively. Others have reported adaptation to BC from 2 ppm in WT strains to 10 ppm MIC in adapted strains (Yu et al., 2018). While these adaptations achieved a higher MIC than was observed in the present study, additional components of the formulated commercial QAC product may have inhibited the adaptation from progressing to BC-adaptation levels. In fact, the majority of published studies on QAC and *Listeria* spp. have been performed using BC as opposed to commercial sanitizer preparations, such as the cQAC used in this study. BC contains an alkyl chain length distribution from C8 to C18. Previous research using narrower alkyl chain lengths has determined that Gram-positive bacteria, such as *L. monocytogenes*, are most affected at chain lengths of C12–C14 whereas longer chain lengths of C14–C16 are more effective against Gram-negative bacteria, such as *Escherichia coli* (Gilbert and Moore, 2005). Commercial QAC products have been formulated to contain QACs with a specific distribution of alkyl chain lengths (e.g., C14: 50%, C12: 40%, and C16: 10%) to optimize antimicrobial activity. These may also be formulated with additional ingredients, including divalent chelators like EDTA, that assist in destabilizing the bacterial cell membrane structures (Gilbert and Moore, 2005). The distribution of alkyl chain lengths and other additives in the commercial product formulation likely influence adaptation and complicate comparing MIC and MBC values from independent studies.

The primary mechanism of QAC adaptation and tolerance is largely attributed to the presence and upregulation of specific efflux pumps (Aase et al., 2000; Romanova et al., 2006; Kovacevic et al., 2013; Yu et al., 2018). Previous studies have reported the assistance of QAC specific efflux systems playing a role in resistance or adaptation, specifically the *bcrABC* cassette (Elhanafi et al., 2010; Dutta et al., 2013), *emrE* (Kovacevic et al., 2016), *emrC* (Kremer et al., 2017), *qacC* and *qacH* (Müller et al., 2013). cQAC tolerance of the adapted strains in the present study was lost with the addition of reserpine, indicating that efflux systems are playing a significant role in the experimentally achieved cQAC tolerance. While the previously reported genomic analyses of six WT strains examined here (Bland et al., 2021) revealed the lack of efflux pumps commonly linked to increased tolerance to QAC (e.g., *bcrABC*, *qacC*, *qacH, emrE*, or *emrC*), the restoration of the WT phenotype in the presence of reserpine, a known efflux pump inhibitor, suggests the role of other efflux pumps in the cQAC adaptation in these isolates.

Research studies have reported that two efflux pumps, *mdrL* and *lde*, ubiquitous in *L. monocytogenes* (Romanova et al., 2006; Yu et al., 2018), are upregulated following the exposure or adaptation to QAC, suggesting their role in tolerance toward the compound. Yu et al. (2018) found that the relative expression of *mdrL* was significantly higher in six *L. monocytogenes* strains following adaptation to a QAC. In contrast, Jiang et al. (2018) reported that MICs were not affected by the absence of *lde* in a deletion mutant. While it can be speculated that *mdrL* and *lde* may have played a role in adaptation, as they were both present in our studied isolates, there are additional, more broadly found multidrug resistant (MDR) efflux systems in Gram-positive bacteria that may be assisting in tolerance and cross-resistance (Tamburro et al., 2015; Schindler and Kaatz, 2016; Meier et al., 2017; Du et al., 2018). Some of these major families of MDR efflux systems have been described by Schindler and Kaatz (2016) including: (1) ATP-binding cassette, (2) major facilitator superfamily (*lde*), (3) multidrug and toxic extrusion systems (*mdrL*), (4) small multidrug resistance, and (5) resistance nodulation cell division (RND) family.

Other mechanisms of QAC tolerance have been explored in isolates without known efflux pumps associated with QACs. In a study by Meier et al. (2017) 45 *L. monocytogenes* isolates were found to possess high tolerance to BC (MIC > 20 ppm). When exposed to reserpine, only four isolates exhibited reduced tolerance to BC. They suggested that the majority of their BC-tolerant strains did not rely on efflux systems. Studies have suggested that the decrease in cell membrane permeability plays an important role in the reduction of tolerance to QACs (McDonnell and Russell, 1999; To et al., 2002; Wessels and Ingmer, 2013) and chlorine and selective antibiotics (Bansal et al., 2018). An increase in the size (cells were elongated and filamentous) and a shift in the fatty acid composition of the cell (shift from shorter to longer fatty acid following adaptation-resulting in decrease in fluidity of the cell), was reported following BC adaptation, suggesting changes in the cell structure/membrane as a possible tolerance mechanism (To et al., 2002). While outside the scope of the data presented here, due to the lack of major efflux systems that have previously been linked to assisting in QAC tolerance development, it would be of interest to consider changes in cellular permeability and fatty acid composition in our isolates as a possible mechanism aiding the adaptation and cross resistance process.

Following cQAC adaptation, we observed a shift in susceptibility to CIP, KAN, NOV, PEN, and to a lesser degree CLI and CHL amongst the six *L. monocytogenes* strains tested. In previous studies, *L. monocytogenes* adapted to QAC resulted in phenotypic changes comparable to those seen in the present study with some minor differences. Similar to our results, the shift in susceptibility toward CIP following QAC adaptation has been described by Rakic-Martinez et al. (2011) and Yu et al. (2018), who both reported an increase in MIC of CIP following adaptation to BC. The decreased susceptibility to KAN following BC adaptation reported by Romanova et al. (2006) for 3/4 tested isolates is similar to what we observed here, with the shift from sensitive to intermediate AMR profiles in 3/6 isolates. In contrast, Yu et al. (2018) did not see any changes in susceptibility to KAN in BC-adapted isolates. These data suggest that the adaptive mechanisms for KAN differ among isolates.

Quaternary ammonium compound and CIP tolerance/resistance has been previously reported (Rakic-Martinez et al., 2011; Kovacevic et al., 2013), and appears to be bidirectional, where isolates adapted to QAC become more resistant to CIP and isolates adapted to CIP become more tolerant to QAC. CIP is a fluoroquinolone; its mechanism of action involves preventing DNA separation prior to cell division. Resistance to quinolones in Gram-positive bacteria is typically due to mutations in the intercellular targets of the compound, *gyrA* and *gyrB* (DNA gyrase), *parC* and *parE* (topoisomerase IV) or decreased uptake into the cell via efflux pumps (Lampidis et al., 2002; Jiang et al., 2018). Jiang et al. (2018) found no mutations in the genes targeted by quinolones. However, transcription levels of *lde* increased across the four CIP adapted mutants, suggesting that the resistance development was largely due to decreased uptake into the cell (Jiang et al., 2018). Others have suggested that point mutations in *fepR* (regulator for *fepA*) are in part responsible for resistance to CIP (Wilson et al., 2018). FepA, a part of the multidrug and toxic compound extrusion (MATE) family of efflux pumps, is regulated by *fepR* (Guerin et al., 2014). When MICs of various antibiotics of WT *L. monocytogenes* and its *fepR* deletion mutant were compared, Guerin et al. (2014) reported susceptibility changes only in fluroquinolones. They also saw a change in BC susceptibility in the *fepR* deletion mutant, with an MIC increase from 4 to 8 ppm, suggesting that overexpression of the *fepA* efflux pump may play a role in BC tolerance. An Australian study by Wilson et al. (2018) found 2/100 *L. monocytogenes* possessing CIP resistance; in one of the isolates CIP resistance was linked to a mutation at nucleotide position 181 in *fepR*, resulting in PMSC. A point mutation in *fepR* was also seen in the other CIP resistant isolate, at nucleotide position 170, leading to an amino acid change (alanine to glycine). However, this mutation did not result in a PMSC, but rather this isolate possessed a full length *fepR* (Wilson et al., 2018). Similarly, our results found point mutations at different locations in *fepR*, some of which resulted in a PMSC and others that did not. In our isolates, both the location of the mutation as well as the resulting PMSC were downstream of what was previously seen by Guerin et al. (2014) and Wilson et al. (2018), resulting in a slightly longer gene. The multiple and independent mutations in *fepR* in the current study are strong evidence that loss of *fepR* function is important for *L. monocytogenes* adapting to cQAC. However, definite proof of the role of *fepR* and the level of its contribution to QAC adaptation would require deletion and complementation mutants.

Previous studies evaluating cross-resistance of QAC and antibiotics amongst *L. monocytogenes* have not, to our knowledge, evaluated NOV. NOV is less commonly used due to its decreased efficacy, but it has synergistic activity with tetracyclines, and can be used as an alternative to penicillin (May et al., 2017). Our results suggest that there is a relationship between adaptive QAC tolerance and NOV resistance, as a shift in susceptibility was seen across all six isolates. In particular, a dramatic shift from susceptible to resistant classification was observed in two strains, WRLP354 and WRLP530. Similar to quinolones, NOV inhibits *gyrB* subunit of the bacterial DNA gyrase enzyme involved in energy transduction (Hof et al., 1997; May et al., 2017). The phenotypic cross-resistance can be associated with mutations in the target for NOV or changes in cellular structure to reduce accumulation within the cell. In Gram-positive bacteria, such as *Bacillus lincheniformis*, morphological changes have been associated with NOV resistance. Specifically, in the presence of NOV the cells grow as long filaments as opposed to long chains (Robson and Baddiley, 1977). In Gram-negative bacteria, NOV resistance has been associated with a two-component regulatory system (*baeSR)*, activating an efflux pump system (*mdtABC)*. This is believed to lead to reduced NOV accumulation within the cell (Baranova and Nikaido, 2002; Nagakubo et al., 2002). In our isolates, we did not observe mutations in *gyrAB*. While morphological changes were not investigated, they cannot be ruled out as potential mechanisms aiding the resistance (Robson and Baddiley, 1977).

Our results also indicate that cQAC adaptation affects PEN resistance, with decreased zones of inhibition observed. PEN is a ß-lactam antibiotic, similar to AMP. However, unlike AMP, which is frequently used in the treatment of listeriosis, PEN is not typically used due to increasing reports of microbial resistance (Wilson et al., 2018). The isolates tested in this study were all classified as either having intermediate or resistant PEN profiles. However, while we observed a slight decrease in the zone of inhibition for AMP following cQAC adaptation, all tested isolates remained well within the susceptible classification (Figure 2). ß-lactam antibiotics, such as PEN, work to inhibit cell wall biosynthesis through interactions with penicillin-binding proteins (PBP). *Listeria* spp. have five PBP, and PBP3 is largely the target of ß-lactams (Vicente et al., 1990; Hof et al., 1997). Insertional mutagenesis in associated genes in *L. monocytogenes* EGDe (e.g., *lmo0441*, *lmo0504*, *lmo1438*, and *lmo2229*) resulted in acquired resistance toward PEN (Guinane et al., 2006). In *S. pneumoniae*, PEN resistance typically occurs from recombination events leading to mosaic PBP, which in turn can lead to reduced affinity for PEN and diminished effect on the cell (Blair et al., 2015).

It has been reported that exposure to QAC can increase the expression of virulence genes, such as *prfA* and *inlA* (Kastbjerg et al., 2010). Since cQAC adaptation impacted AMR profiles and led to genomic changes in some of our studied isolates, it was prudent to explore if virulence genes were affected, especially since our isolates belong to hypervirulent clonal complexes (Maury et al., 2016). While we did not look at the expression of virulence genes following cQAC adaptation, it is of note that no mutations in *L. monocytogenes* virulence genes were observed following cQAC adaptation.

## Conclusion

Collectively, our data demonstrate the potential for *L. monocytogenes* isolates to develop cross-resistance between a cQAC and antibiotics representing different classes. While the increased MIC and MBC of cQAC-adapted isolates remained well below the manufacturer recommended concentration for the commercial product, it is not uncommon for bacterial cells to be exposed to lower or sublethal concentrations of sanitizers in the processing environment either by dilution, presence of organic matter (e.g., decreasing the efficacy of the sanitizer), or issues with the hygienic design of equipment or facility (e.g., resulting in microbial niches, biofilm formation or dilution effect of sanitizer). The isolates in the present study were adaptable to 1 ppm higher than the WT MIC. This minimal MIC increase suggests that adaptation of *L. monocytogenes* to a degree that would render the cQAC product ineffective is not likely in commercial settings. However, the present study was done with planktonic cells and so the additional tolerance and protection provided in biofilms, both in terms of initial susceptibility and degree of adaptation, likely differs. In a processing facility, the circumstances that may allow for exposure to minimal concentrations of cQAC are often associated with improper cleaning and inadequate sanitation practices that may not be addressing microbial niches. This can lower the effectiveness of the sanitizer or may be resulting in a dilution effect in some areas of the facility. Our data highlight that sublethal exposures to cQACs could have deleterious effects if adapted *L. monocytogenes* strains become implicated in human illness that requires antibiotic treatment. The potential for *L. monocytogenes* to develop cross-resistance to clinically relevant antibiotics following minimal adaptation to a formulated cQAC product is especially concerning. In particular, this trend amongst genotypes that are classified as hypervirulent and frequently involved in cases of listeriosis highlights the need to better understand the effect that sanitizer exposures and low-level adaptations may have on the AMR development and a potential public health risk.

## Data Availability Statement

The dataset presented in this study can be found in the NCBI Sequence Read Archive (SRA) under BioProject PRJNA771688.

## Author Contributions

JW-C and JK: conceptualization. RB and ER: data curation. RB, AW, JW-C, and JK: formal analysis and methodology. JK: funding acquisition and project administration. AW, JC, JW-C, and JK: supervision and writing – review and editing. AW: validation. RB: visualization and writing – original draft. All authors have read and agreed to the published version of the manuscript.

## Conflict of Interest

The authors declare that the research was conducted in the absence of any commercial or financial relationships that could be construed as a potential conflict of interest.

## Publisher’s Note

All claims expressed in this article are solely those of the authors and do not necessarily represent those of their affiliated organizations, or those of the publisher, the editors and the reviewers. Any product that may be evaluated in this article, or claim that may be made by its manufacturer, is not guaranteed or endorsed by the publisher.
