# Supplementary material for: Adaptation to a Commercial Quaternary Ammonium Compound Sanitizer Leads to Cross-Resistance to Select Antibiotics in Listeria monocytogenes Isolated From Fresh Produce Environments
Source: Front Microbiol. 2022 Jan 10;12:782920. doi: 10.3389/fmicb.2021.782920 (PMC8784610; doi:10.3389/fmicb.2021.782920)
Supplement: Supplementary file 1 [file Table_1.DOCX]

Supplementary Material

**Table S1 |** Antibiotic break points for a panel of 17 antibiotics used to assess antimicrobial resistance of *L. monocytogenes* using disk diffusion assay.

| Antibiotic | Abbreviation | Concentration (µg) | Zone of Inhibition | | |
| --- | --- | --- | --- | --- | --- |
|  |  |  | **Resistant (<mm)** | **Intermediate (mm)** | **Sensitive (>mm)** |
| Amikacin | AMK | 30 | 14 | 15-16 | 17 |
| Ampicillin | AMP | 10 | 19 | -^a^ | 20 |
| Cefoxitin | FOX | 20 | 14 | 15-17 | 18 |
| Chloramphenicol | CHL | 20 | 12 | 13-17 | 18 |
| Ciprofloxacin | CIP | 5 | 15 | 16-20 | 21 |
| Clindamycin | CLI | 2 | 14 | 15-20 | 21 |
| Erythromycin | ERY | 15 | 14 | 15-22 | 23 |
| Gentamicin | GEN | 10 | 12 | 13-14 | 15 |
| Imipenem | IMP | 10 | 13 | 14-15 | 16 |
| Kanamycin | KAN | 30 | 13 | 14-17 | 18 |
| Novobiocin | NOV | 30 | 13 | 18-21 | 22 |
| Penicillin | PEN | 10 U^b^ | 19 | 20-27 | 28 |
| Rifampin | RIF | 5 | 16 | 17-19 | 20 |
| Streptomycin | STR | 10 | 11 | 12-14 | 15 |
| Co-trimocazole^c^ | SXT | 1.25/23.75 | 10 | 11-15 | 16 |
| Tetracycline | TET | 30 | 14 | 15-18 | 19 |
| Vancomycin | VAN | 5 | 9 | - | 10 |

^a^No intermediate breakpoints, only sensitive or resistant (-).

^b^Penicillin disk concentration in international units of penicillin (U).

^c^Co-trimoxazole is composed of two antibiotics, trimethoprim (1.25 µg) and sulfamethoxazole (23.75 µg).
